# Tissues Use Resident Dendritic Cells and Macrophages to Maintain Homeostasis and to Regain Homeostasis upon Tissue Injury: The Immunoregulatory Role of Changing Tissue Environments

**DOI:** 10.1155/2012/951390

**Published:** 2012-12-03

**Authors:** Maciej Lech, Regina Gröbmayr, Marc Weidenbusch, Hans-Joachim Anders

**Affiliations:** Medizinische Klinik und Poliklinik IV, Klinikum der Universität München (LMU), Ziemssenstrasse 1, 80336 München, Germany

## Abstract

Most tissues harbor resident mononuclear phagocytes, that is, dendritic cells and macrophages. A classification that sufficiently covers their phenotypic heterogeneity and plasticity during homeostasis and disease does not yet exist because cell culture-based phenotypes often do not match those found *in vivo*. The plasticity of mononuclear phagocytes becomes obvious during dynamic or complex disease processes. Different data interpretation also originates from different conceptual perspectives. An immune-centric view assumes that a particular priming of phagocytes then causes a particular type of pathology in target tissues, conceptually similar to antigen-specific T-cell priming. A tissue-centric view assumes that changing tissue microenvironments shape the phenotypes of their resident and infiltrating mononuclear phagocytes to fulfill the tissue's need to maintain or regain homeostasis. Here we discuss the latter concept, for example, why different organs host different types of mononuclear phagocytes during homeostasis. We further discuss how injuries alter tissue environments and how this primes mononuclear phagocytes to enforce this particular environment, for example, to support host defense and pathogen clearance, to support the resolution of inflammation, to support epithelial and mesenchymal healing, and to support the resolution of fibrosis to the smallest possible scar. Thus, organ- and disease phase-specific microenvironments determine macrophage and dendritic cell heterogeneity in a temporal and spatial manner, which assures their support to maintain and regain homeostasis in whatever condition. Mononuclear phagocytes contributions to tissue pathologies relate to their central roles in orchestrating all stages of host defense and wound healing, which often become maladaptive processes, especially in sterile and/or diffuse tissue injuries.

## 1. Introduction

Mononuclear phagocytes are a group of phenotypic distinct members, often referred to as either macrophages or dendritic cells (DC), that derive from myeloid precursors and that contribute to the functions of peripheral tissues [1]. During the last decades, research has focused on the cell-type-specific properties of these cells in culture, which then led to an *immunocentric* view of their role in disease like if they were primed like T cells to infiltrate target organs to cause tissue damage and drive progressive scaring [2, 3]. A more *tissue-centric* view of these processes, claiming that the tissues define phenotype and function of resident and infiltrating immune cells to meet tissues needs during homeostasis and disease, seems provocative [4, 5]. In this paper we apply the *tissue-centric* perspective to discuss the role of resident and infiltrating macrophages and dendritic cells in different organs. We examine tissue needs to maintain homeostasis and how to regain homeostasis upon tissue injury. Furthermore, we discuss how published data supports the view that changing tissue environments induce the well-known different phenotypes of mononuclear phagocytes, a process that not only enforces each of the different environments but also explains the contribution of these cells to the different tissue pathologies. This slightly different perspective may somewhat shape our understanding of macrophage heterogeneity and tissue pathology but certainly also raise new questions for future research.

## 2. Tissues Need Mononuclear Phagocytes to Maintain Homeostasis

**Figure 1:**
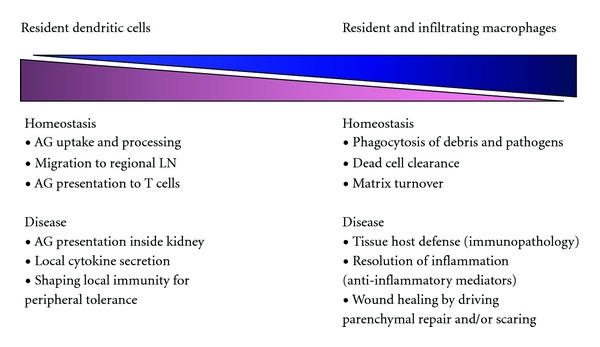
Roles of resident dendritic cells and tissue macrophages in homeostasis and disease. AG: antigen; LN: lymph nodes.

All solid organs and most other tissues harbor a network of DC or macrophages (Table 1). Due to their considerable plasticity and heterogeneity, the tissue-based DC and macrophage populations have been defined as mononuclear phagocytes [1, 6, 7]. These cells provide several important physiological functions during homeostasis (Figure 1). For example, organs like the lung and the liver are exposed to pathogen components from the air or from the gut barrier, respectively, which explains the predominance of a macrophage phenotype that has a higher capacity for phagocytic clearance of pathogen components. The same applies to the bone marrow that requires macrophages for the clearance of the nuclei that get expelled from erythroblasts during their maturation towards erythrocytes [8]. In contrast, the gut mucosa hosts dendritic cells that turn signals from the intestinal flora into the secretion of mitogenic mediators that assist in maintaining an intact epithelial lining of the gut as an important component of the intestinal barrier function [2]. Sterile organs rather harbor dendritic cells. During homeostasis, dendritic cells are sensors and guardians of peripheral tolerance due to their capacity to process self-antigens and signal tolerance to the T-cell pool upon evading the peripheral organs via the lymphatics to reach regional lymph nodes [9]. This functional property constantly assures the quiescence of the immune system in homeostasis. Dendritic cells share certain functions with tissue macrophages such as particle phagocytosis and danger recognition/signaling upon the recognition of pathogens, hence these cells taken together are now referred to as the mononuclear phagocyte system.

## 3. Tissues Need Mononuclear Phagocytes to Fight Threats to Homeostasis

**Figure 2:**
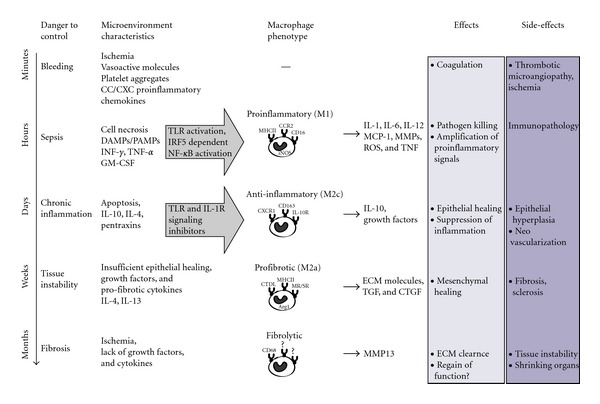
Tissue microenvironments and predominant macrophage phenotypes. Danger control involves several response programs that operate from seconds to months after injury. In each of these phases, the tissue environment shapes the phenotype of resident and infiltrating mononuclear phagocytes, which then enforce the particular environment in a feed-forward loop. Their potential to amplify inflammation, healing, or scaring has consequences on the tissue that may be beneficial or unfortunate in terms of rapidly regaining homeostasis and full function of the organ. This illustrates that the evolutionary programs of danger control are not perfect in all settings, but the fact that they were positively selected during evolution allows only one interpretation: they obviously represented the best compromise between the different needs of multicellular organisms. Where these programs cause malfunction, also mononuclear phagocytes contribute to the “disease” process. TLR: Toll-like receptor, ROS: reactive oxygen species, and ECM: extracellular matrix.

**Figure 3:**
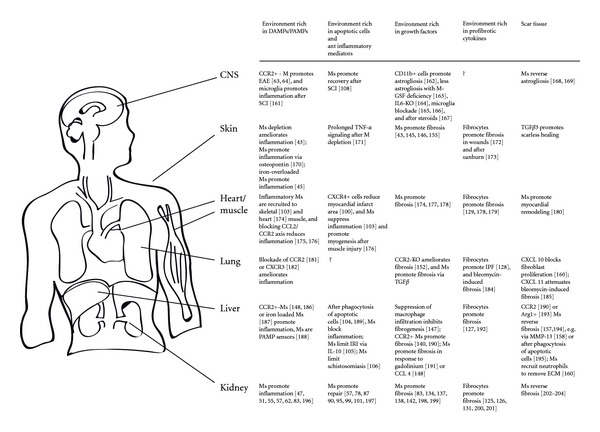
Macrophages in different phases of solid organ pathologies.

Tissue injury can be traumatic, infectious, toxic, ischemic, or autoimmune to which the tissue responds by a set of evolutionary conserved danger response programs (Figure 2) [18]. Traumatic injury usually involves vascular injury, which immediately activates clotting to control the danger of potentially fatal bleeding. Inflammation is the second danger response program that is needed to avoid pathogen entry to control infections [2]. Pathogens release pathogen-associated molecular patterns (PAMPs), and damaged tissue cells release damage-associated molecular patterns (DAMPs). PAMPs and DAMPs have an identical capacity to ligate Toll-like receptors (TLR) and other pattern recognition receptors on immune and nonimmune cells in the tissue to secrete proinflammatory cytokines and chemokines [19–21] (Figure 2). DAMPs may originate from intracellular sources that get released by cell necrosis, such as histones [22], HMBG1 [23], ATP [24], or uric acid [25]. Furthermore, proinflammatory macrophages release matrix metalloproteinases (MMPs) and hyaluronidase that digest extracellular matrix (ECM) proteins and thereby reduce the ECM viscosity. This process, together with increased vascular permeability, induces tissue swelling, promotes leukocyte migration, and increases the accessibility of surface receptors to their PAMP and DAMP agonists, that is, inflammation. It is of note that ECM digestion produces small ECM peptides and glycosaminoglycans, which can turn into immunostimulatory DAMPs that enhance the local proinflammatory microenvironment [26]. Tamm-Horsfall protein/uromodulin is another example for a compartment-specific DAMP. It is exclusively secreted at the luminal membrane of distal tubular epithelial cells into the urinary compartment of the tubular lumen. During tubular injury, it may lack into the renal interstitium, where it has the capacity to activate intarenal mononuclear phagocytes via TLR4 and the NLRP3 inflammasome [27, 28]. This way, traumatic and infectious injuries induce a PAMP- and DAMP-rich tissue environment that gets reenforced by dendritic cell and macrophage activation (Figures 2 and 3) [29, 30]. Activation of innate immunity subsequently involves the recruitment of additional leukocytes from the circulation including monocytes as well as IFN-*γ*-secreting NK cells to the injured tissue. When, upon arrival, the infiltrating macrophages get exposed to the PAMP- and/or DAMP-rich environment, hence, this will lead to their full activation towards the proinflammatory macrophage phenotype [20, 31–33]. Polarization to classically activated macrophages requires interferon-related factor (IRF)5 [34]. Such macrophages secrete IL-1, IL-12, IL-23, TNF-*α*, and ROS and induce iNOS, MHCII^hi^, and IL1-R, an expression profile that was classified as “M1” classically activated macrophage by *in vitro* stimulation with IFN-*γ*, TNF-*α*, LPS, or GM-CSF [31]. Polarization towards this bactericidal macrophage type provides the tissue with efficient support for local host defense against pathogens. This potentially life-saving effector functions outweigh the unspecific toxicity of the secreted mediators that can cause significant immunopathology and even transient organ dysfunction (Figure 2) [31, 35]. 

**Figure 4:**
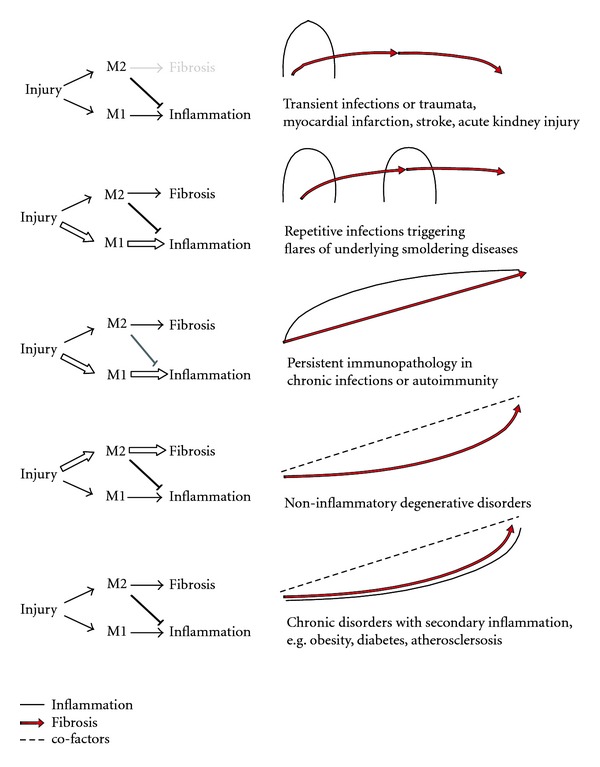
Translating the paradigm of classically activated (M1) and alternatively activated (M2) macrophage into clinical contexts. Classically activated (M1) macrophages promote tissue inflammation and immunopathology based on their role in host defense against intracellular pathogens. Extracellular pathogens are mostly attached by humoral factors such as complement, but when they persist, alternatively activated (M2) macrophages provide means of host defence that involve anti-inflammatory, progenerative, and profibrotic elements. The balance of inflammation and fibrosis varies over time and is different in different disease states and often operates in parallel. For this reasons tissue biopsies often become difficult to read and display a mixture of all these elements. The figure provides examples of common disease entities to illustrate how changing tissue environments involve M1- and M2-macrophages-mediated pathology either in a sequential manner, in an intermittent manner, or in a parallel manner, which largely depends on the associated underlying disease processes and cofactors. We propose that the sequential pattern shown at the top was the one that dominated during the evolution of wound healing from the stage of the first multicellular organisms, for example, healing of mechanical trauma in nonsterile environments. We further propose that all other mixtures that doctors often get to see in pathology textbooks and in their clinics originate from that and represent maladaptive variants of this underlying danger response program that was otherwise extremely successful during evolution.

The danger response program of classically-activated mononuclear phagocyte-driven tissue inflammation for host defense remained evolutionally conserved in sterile solid organ injuries [36–38]. However, DAMP-driven proinflammatory macrophage effects are not needed to kill pathogens and mostly cause unnecessary immunopathology (“collateral damage”). In DAMP-rich but pathogen-free sterile inflammation (ischemic, toxic, or autoimmune injuries), however, this otherwise beneficial response turns into a maladaptive process, with immunopathology that is not balanced by any significant benefit for the tissue [39]. In sterile injuries, the inflammatory phase can be short-lasting, for example, after a transient insult such as a transient ischemia or toxin exposure (Figure 4) [40]. By contrast, inflammation persists upon repetitive or ongoing ischemia or toxin exposure. For example, proton pump inhibitors accelerate gastric and duodenal ulcer healing, also because they reduce persistent acidic damage of the gastric or duodenal mucosa, a process that is required for the resolution of the inflammatory response and for the completion of the wound healing process [41]. As another example, fetal dermal wound healing takes place in a sterile environment without PAMP exposure to the wound. Therefore, much less proinflammatory macrophages are recruited to the site of injury, which, together with the higher regenerative capacity of fetal tissues, explains why fetal wounds heal faster and without scaring [42]. During the early phase of injury, proinflammatory macrophages are entirely dispensable in sterile wounds as their depletion limits the inflammatory response and fastens the healing process [43]. That is why sterile (PAMP-free) wound care is a validated therapeutic strategy to limit the inflammatory response and to enforce healing of surgical wounds or other skin injuries [44]. In addition, in wounds with vascular lesions and subcutaneous bleeding, erythrocyte-derived iron serves as a DAMP that induces the inflammatory macrophage phenotype, which then again suppresses the wound healing process [45, 46]. 

The uselessness of inflammation in sterile injuries provides the rationale for anti-inflammatory and immunosuppressive treatments. For example, inhibiting the recruitment or activation of proinflammatory mononuclear phagocytes drastically reduces immunopathology and organ malfunction in acute and chronic tissue injuries, for example, in a variety of kidney diseases such as in anti-GBM glomerulonephritis [47], lupus nephritis [48–53], antigen-induced immune complex glomerulonephritis [54], renal allograft injury [55], ischemia reperfusion injury [40, 56–58], and adriamycin nephropathy [59]. In addition, environmental factors can aggravate tissue injury by activating mononuclear phagocytes towards a classically activated phenotype [60]. These can be circulating PAMPs, for example, during transient infections (Figures 2 and 4), vaccines or other drugs with distinct immunostimulatory properties. For example, the chemokine antagonists Met-RANTES and AOP-RANTES block monocyte recruitment but still activate resident tissue macrophages, which is sufficient to aggravate preexisting immune complex glomerulonephritis [54]. In contrast, chemokine antagonists without this immunostimulatory side effect were shown to substantially reduce the related immunopathology in multiple disease models of the kidney [61, 62], the CNS [63, 64], the liver, and other noninfectious types of solid organ inflammation as listed in Figure 3. 

Dendritic cells support host defense by rather leaving the tissue via the lymphatics to carry foreign antigens to the regional lymph node, which then trigger antigen-specific immune responses and the influx of antigen-specific effector cells that contribute to tissue inflammation. The PAMP-driven innate immunity strongly activates this process in an adjuvant-like manner. Hence, PAMP exposure, for example, during transient infections, can induce the onset or flares of subclinical or chronic autoimmune disorders (Figure 4) [49–54, 65–69]. 

Together, injuries change the homeostatic tissue towards DAMP- and or PAMP-rich environments which activate resident and infiltrating mononuclear phagocytes. These produce additional immunostimulatory mediators that setup local inflammation, a process that is evolutionally conserved to control invading pathogens. This danger response program is often associated with significant immunopathology, especially in sterile inflammation. This causes unnecessary tissue injury and becomes a maladaptive disease pathomechanism, which provides the rationale for immunosuppressive and anti-inflammatory therapies.

## 4. Tissues Need Mononuclear Phagocytes to Avoid Excessive Immunopathology and to Orchestrate Repair

Overshooting systemic immune activation holds the risk of death like in early sepsis [70]. Similarly, overshooting organ inflammation holds the risk of acute organ dysfunction like in stroke, myocardial infarction, acute kidney injury, or severe pneumonia. As a consequence, numerous anti-inflammatory mediators provide a balance to immunostimulatory factors, a process that also allows the resolution of inflammation upon pathogen clearance [71–73]. Resolution of inflammation is initiated by a shift in the tissue microenvironment. For example, the early neutrophil influx into a PAMP-rich environment and DAMP release from necrotic cells can change once pathogen control is achieved, so that tissue environments display less PAMPs and DAMPs but become dominated by increasing numbers of apoptotic neutrophils. Macrophage clearance of apoptotic cells is already an important element of peripheral tolerance during homeostasis in healthy tissues, but it becomes an element of the resolution of inflammation in disease [71, 72]. Neutrophil phagocytosis triggers macrophage deactivation and the expression of anti-inflammatory mediators and growth factors that have the potential to stimulate tissue healing [74, 75]. In fact, apoptosis of activated neutrophils and T cells is a mechanism that prevents inappropriate or persistent immunopathology [74]. This also applies to the postinflammatory phase of sterile injuries (Figure 3). For example, transient ischemia reperfusion is associated with cell necrosis and DAMP release followed by the influx of neutrophils and classically activated macrophages for 1–3 days [40]. The excessive phagocytosis of apoptotic neutrophils activates the monocytic phagocytes to release TGF-*β* and IL-10 [76]. Serum amyloid-P, also named pentraxin-2, opsonizes apoptotic cells which further promotes the anti-inflammatory macrophage phenotype [77]. Infiltrating regulatory T cells also produce IL-10 and TGF-*β*, which further supports the polarization towards anti-inflammatory macrophages and also suppresses of T effector cells [78]. This deactivation of proinflammatory macrophages involves the transcription factor IRF4, which competes with IRF5, a nonredundant element of TLR and IL-1R signaling [79–82]. IRF4-deficiency does not allow this phenotype switch [80], hence, persistently activated macrophages contribute to ongoing immunopathology [83]. As another mechanism that promotes resolution of tissue injury, tissue dendritic cells produce pentraxin-3, which then blocks P selectin on the luminal surface of vascular endothelial cells, which blocks further immune cell recruitment [84–86].

The current macrophage classifications are derived from decent *in vitro* study conditions that have not yet integrated apoptotic cells as a stimulus of differentiation [31, 87–92]. However, the M2c phenotype of macrophages stimulated with IL-10 and TGF-*β* display certain characteristics of anti-inflammatory tissue macrophages (Figure 3) [31, 87–92]. The fact that M2c macrophages themselves produce large amounts of IL-10 illustrates how macrophages can amplify their surrounding environments by secreting similar cytokines in a feed-forward loop [93]. These cells are needed to enforce the resolution of inflammation, which is required to tip the balance of host defense and repair towards tissue regeneration (Figure 4). To enhance the regeneration process, anti-inflammatory macrophages acquire a phenotype of growth factor-producing cells that now actively drive epithelial or parenchymal repair. For example, macrophage depletion during the postinflammatory phase of sterile wounds delays wound healing and supports hemorrhage because of a persistent apoptosis of endothelial cells and detachment of the neuroepithelium [43, 94]. In addition, postischemic acute kidney injury involves the phenotypic switch from proinflammatory towards anti-inflammatory macrophages, a process driven by factors released by dying tubular epithelial cells and by the phagocytosis of apoptotic neutrophils [57, 95]. IRF4 or IRAK-M deficiency prevents this phenotype switch, which supports ongoing disease activity in a number of acute and chronic disease states [80, 83, 96–98]. In addition, treatment with recombinant IL-4/IL-10 or genetically modified or transfused IL-10-stimulated macrophages helps to resolve renal inflammation [87–90, 99]. The same phenomenon improves cardiac remodeling after myocardial infarction [100]. Glucocorticoids suppress tissue inflammation by inducing the anti-inflammatory phenotype of tissue macrophages [101, 102]. Monocyte recruitment to skeletal muscle may initially result in a proinflammatory macrophages phenotype, which then rapidly change their phenotype into anti-inflammatory macrophages that assist myogenesis and macrophage depletion that leads to a significantly reduced diameter of regenerating muscle fibers [103]. Toxic liver disease is another example of sterile organ dysfunction. CCl_4_ induces hepatocyte apoptosis and subsequent phagocytic clearance by Kupffer cells, a mechanism that suppresses liver inflammation [104]. Ischemia-reperfusion injury of the liver is associated with significant IL-10 expression, which was found to be crucial for the anti-inflammatory capacity of Kupffer cells [105]. In experimental schistosomiasis, IL-4R*α*-deficiency of macrophages was sufficient to cause a lethal septic phenotype [106], which demonstrates the role of anti-inflammatory cytokines produced by alternatively activated macrophages in the gut and the liver, respectively [107]. Finally, axonal regeneration after spinal cord injury depends on the recruitment of IL-10-producing macrophages to the CNS [108]. 

The anti-inflammatory macrophage phenotype does not only contribute to the resolution of inflammation and the healing phase upon tissue injuries. Also non-necrotic environments of solid tumors induce alternative macrophage activation which then enforces tumor growth [109]. The same applies to degenerative tissue lesions or tissue damage upon slowly accumulating toxins dominated by apoptotic cell death [75].

## 5. Tissues Need Mononuclear Phagocytes for Effective Scaring When Epithelial or Parenchymal Healing Remains Incomplete

Evolution has maintained tissue scaring for its benefits for the function and survival of organisms. Scaring is necessary in more complex multicellular organisms when traumatic amputation or otherwise significant loss of tissue cannot be rapidly regenerated, a process that requires sealing and mechanical stabilization to assure function and survival. For example, a limited pericyte proliferation can assist vascular stability during regeneration upon injury [110]. However, myofibroblast proliferation and extensive fibrosis offer structural benefits only upon focal wounding and strongly depend on the site or compartment of injury. In diffuse fibrosis of the skin, like in progressive scleroderma, holds the potential to destroy the organ, a functional consequence that applies especially to organs that are commonly affected by diffuse injuries such as the lung, the liver, and the kidney [18, 111, 112]. But instead of taking fibrogenesis as a mechanism of progressive organ, destruction fibrous tissue mainly replaces lost parenchyma; therefore, inhibiting fibrogenesis may not necessarily be able to restore tissue function unless being accompanied by significant regeneration of the parenchyma. Therefore, apart from the healing of tendons, bones, and fasciae, only insufficient healing of epithelial and vascular structures is commonly associated with mesenchymal healing, that is, fibrosis when (1) the damage goes beyond epithelial layer injury, which can occur in some organs like skin, intestinal tract, pancreas and other glands or kidney. Damage to mesenchymal cell structures is more complex and requires more time, for example, in bone, tendons, heart, and skeletal muscle. (2) Local progenitor cells do not survive the injury phase. If at all terminally differentiated cells can divide is questionable and the concept of their dedifferentiation for mitotic repair remains under debate [113–120]. The evolving concept that terminally differentiated cells mostly regenerate from the division of committed local progenitor cells in all organs is appealing and could explain why regeneration remains insufficient when these cells get lost during a severe injury phase or undergo senescence, for example, during aging. (3) Repair is compromised by ongoing PAMP or DAMP exposure like during local infection or by persistent or remitting injuries that impair the repair process by persistent inflammation (Figure 4) [103]. 

An insufficient repair creates a microenvironment that becomes dominated by the persistent expression of multiple profibrotic cytokines [44, 94, 121]. In such environments, mononuclear phagocytes become a major source of profibrotic cytokines [3]. *In vitro*, IL-4 and IL-13 induce STAT6 signaling, which induces a macrophage phenotype that predominately releases fibronectin and other ECM molecules and that expresses mannose and scavenger receptors, IL-1R11, FIZZ, and YM-1, that is, M2a macrophages [31]. It remains to be determined whether anti-inflammatory and profibrotic macrophages clearly represent two different types of cells also *in vivo*, because macrophage plasticity usually creates a mixture or continuous variant shifts during tissue remodeling (Figure 4) [35]. However, a pro-fibrogenic phenotype of myeloid cells already exists at the level of circulating monocytes, that is, the fibrocyte that shares phenotypic similarities with monocytes and fibroblasts and that can produce large amounts of collagen [122–126], for example, in the liver [127], the lung [128], the heart [129], and in the kidney [60, 125] (Figure 3). However, their quantitative contribution to tissue scaring has been questioned by GFP lineage tracing of collagen 1*α*1-producing cells, that found only a minor contribution of fibrocytes to renal fibrogenesis and scaring [112, 130, 131]. 

Chemokine receptor CCR1 seems to be essential for profibrotic macrophage- and fibrocyte-mediated fibrosis because lack of CCR1 or CCR1 antagonism prevents progressive tissue scaring in many different organs and various types of injuries [12, 132–144]. Macrophages that contribute to dermal fibrosis express CXCR3 [145]. Insufficient macrophage activation in chronic diabetic leg ulcers delays scar formation, which can be restored by administering GM-CSF [146]. Similar mechanisms apply to progressive fibrosis of solid organs (Figure 3). Targeting the MCP-1/CCR2 axis [147, 148] or deficiency in CCR1/CCR5 blocked the recruitment of profibrotic macrophages, which was associated with less liver fibrosis [140] and renal fibrosis [132, 134, 137, 138, 149–151]. In the lung, CCR2 deficiency attenuated bleomycin-induced scaring [152], which was shown to be mediated by IL-13 signaling via IL-13-R*α*1 and IL-13-R*α*2 to stimulate TGF*β* secretion in macrophages [153]. Together, tissues use their resident and infiltrating mononuclear phagocytes to fill the gaps of lost parenchyma, which stabilizes the tissue integrity. This is helpful upon focal injuries but may contribute to tissue loss in diffuse injuries, thus this evolutionally conserved danger control program often becomes a maladaptive disease process, especially when epithelial healing remains insufficient.

## 6. Tissues Need Fibrolytic Mononuclear ****Phagocytes to Clear Excess Extracellular Matrix

Progressive lung fibrosis, renal interstitial fibrosis, or liver cirrhosis is characterized by parenchymal cell loss which gets partially replaced by fibrous tissue. Whether fibrogenesis itself contributes to the loss of parenchymal cells remains under debate [112, 154]. However, it is a matter of fact that even though fibrosis is often associated with advanced disease, it does not always progress to end-stage organ failure [155]. In fact, fibrosis can be a transient process that stabilizes tissue integrity during repair and almost entirely resolves later [44]. For example, dermal wound healing ends in the smallest possible scar, after a skin cut. Evidence for inducible fibrolysis in the skin comes from recombinant TGF*β*-3 application in humans as well as preclinical models [156]. TGF*β*-3 application prevented excessive proliferation of myofibroblasts and scar formation similar to fetal wound healing [156]. 

Macrophages are capable of clearing ECM via the secretion of selected MMPs, a process that limits and potentially reverses fibrosis [156]. For example, scar-associated macrophages remove fibrous tissue that accumulates after toxic liver injury by secretion of MMP13 and by recruiting neutrophils to the scar tissue [157–159]. In addition, such “fibrolytic” macrophages secrete CXCL10, which blocks the proliferation of fibroblasts in bleomycin-induced pulmonary fibrosis [160]. Excessive scaring, obviously, increases the physiological capacity of tissue macrophages to break down ECM during homeostasis into a scar tissue-reducing phenotype. Hence fibrolytic macrophages need to be added to the list of functionally important macrophage phenotypes (Figures 2 and 3). Surface markers that clearly identify fibrolytic macrophages remain to be described. One should keep in mind that MMP-secreting macrophages have been reported to contribute to tissue degradation by chopping up basement membranes [160]. Therefore, the fibrolytic macrophage may also rather contribute to tissue atrophy and further reduce the size and function of a shrunken organ, if its presence is not associated with extensive regeneration of de novo parenchyma. In fibrotic livers, however, transfer of bone marrow-derived macrophages was shown to reverse hepatic fibrosis and to improve regeneration and function of the liver [160].

## 7. Summary and Conclusions

Most tissues host mononuclear phagocytes because they help them to maintain peripheral tolerance. Mononuclear phagocytes provide this support by processing “self” into tolerogenic signals to the immune system (all organs), by removing cell debris (e.g., bone marrow) and incoming pathogen components (e.g., liver), and by turning PAMP recognition into epithelial growth to maintain barriers (e.g., gut). As different tissues have different needs to maintain tolerance, mononuclear phagocytes display very heterogeneous phenotypes already during homeostasis. These phenotypes are a result of the specific environment, which is provided in each organ. Similarly, when tissue injuries alter the organ-specific tissue environment, also the resident as well as the infiltrating myeloid cells will be affected as a result of their plasticity to polarize to different phenotypes in different environments. PAMP- and DAMP-rich (necrotic) environments [161–204] prime proinflammatory monocytic phagocytes for host defense, which, however, involves immunopathology, especially during sterile inflammation. Postinflammatory environments including tumor stroma are dominated by apoptotic cell bodies, which trigger polarization towards anti-inflammatory or tumor-associated macrophages that suppress immunity and support cell growth, which could mean epithelial healing but also tumor growth. A healing tissue environment, especially during insufficient epithelial healing, is dominated by growth factors that prime macrophages towards a profibrotic phenotype secreting profibrotic cytokines and ECM components. Scar tissue is hypoxic and lacks growth factors, which enable fibrolytic macrophages to predominantly secrete proteases that remove ECM. Together, mononuclear phagocytes are amplifiers of their surrounding environments because the tissue primes macrophages according to its needs to stabilize and to reenforce the current environment. Thus, organ- and disease-phase-specific environments determine the associated macrophage and dendritic cell heterogeneity, which assures their support to maintain and regain tissue homeostasis in whatever condition. Pathogenic roles of these cells in diffuse tissue injuries are related to maladaptive wound healing programs. 

## Figures and Tables

**Table 1:** Resident mononuclear phagocytes in various organs and tissues.

Tissue	Macrophages	Dendritic cells
Skin	Dermal macrophages [10]	Dermal DCs, Langerhans cells [10]
Bone	Osteoclasts [10]	
Bone marrow	Bone marrow macrophages [11]	
Ovary/testis	Ovarian macrophages [12]	
Kidney		Interstitial DCs [7, 13]
Pancreas		Dendritic cell precursors [14]
Spleen	Marginal zone macrophages, red pulp macrophages [10]	iDCs, follicular DCs [15]
Liver	Kupffer cells [10]	Plasmacytoid DCs, cDCs [16]
Colon	Intestinal macrophages [17]	Lamina propria DCs [17]
Ileum	Intestinal macrophages [17]	Lamina propria DCs [17]
Stomach	Intestinal macrophages [17]	Lamina propria DCs [17]
Lung	Alveolar macrophages [10]	
Brain	Microglia [10]	

DCs: dendritic cells.
